# A comparison of continuous and bi-level positive airway pressure non-invasive ventilation in patients with acute cardiogenic pulmonary oedema: a meta-analysis

**DOI:** 10.1186/cc4861

**Published:** 2006-03-27

**Authors:** Kwok M Ho, Karen Wong

**Affiliations:** 1 Department of Intensive Care, Royal Perth Hospital, Perth, Western Australia, Australia; 2 School of Population Health and Medicine and Pharmacology, University of Western Australia, Crawley, Perth, Western Australia, Australia

## Abstract

**Introduction:**

We conducted the present study to investigate the potential beneficial and adverse effects of continuous positive airway pressure (CPAP) compared with bi-level positive airway pressure (BiPAP) noninvasive ventilation in patients with cardiogenic pulmonary oedema.

**Method:**

We included randomized controlled studies comparing CPAP and BiPAP treatment in patients with cardiogenic pulmonary oedema from the Cochrane Controlled Trials Register (2005 issue 3), and EMBASE and MEDLINE databases (1966 to 1 December 2005), without language restriction. Two reviewers reviewed the quality of the studies and independently performed data extraction.

**Results:**

Seven randomized controlled studies, including a total of 290 patients with cardiogenic pulmonary oedema, were considered. The hospital mortality (relative risk [RR] 0.76, 95% confidence interval [CI] 0.32–1.78; *P *= 0.52; *I*^2 ^= 0%) and risk for requiring invasive ventilation (RR 0.80, 95% CI 0.33–1.94; *P *= 0.62; *I*^2 ^= 0%) were not significantly different between patients treated with CPAP and those treated with BiPAP. Stratifying studies that used either fixed or titrated pressure during BiPAP treatment and studies involving patients with or without hypercapnia did not change the results. The duration of noninvasive ventilation required until the pulmonary oedema resolved (weighted mean difference [WMD] in hours = 3.65, 95% CI -12.12 to +19.43; *P *= 0.65, *I*^2 ^= 0%) and length of hospital stay (WMD in days = -0.04, 95% CI -2.57 to +2.48; *P *= 0.97, *I*^2 ^= 0%) were also not significantly different between the two groups. Based on the limited data available, there was an insignificant trend toward an increase in new onset acute myocardial infarction in patients treated with BiPAP (RR 2.10, 95% CI 0.91–4.84; *P *= 0.08; *I*^2 ^= 25.3%).

**Conclusion:**

BiPAP does not offer any significant clinical benefits over CPAP in patients with acute cardiogenic pulmonary oedema. Until a large randomized controlled trial shows significant clinical benefit and cost-effectiveness of BiPAP versus CPAP in patients with acute cardiogenic pulmonary oedema, the choice of modality will depend mainly on the equipment available.

## Introduction

Acute cardiogenic pulmonary oedema is a common medical emergency. The majority of patients with acute pulmonary oedema will improve with oxygen and pharmacological therapy. However, assisted ventilation may be needed in patients with severe cardiogenic pulmonary oedema who remain hypoxaemic and in respiratory distress despite conventional medical therapy [1].

Studies have shown that noninvasive continuous positive airway pressure (CPAP) ventilation can improve gas exchange, decrease respiratory and heart rate, reduce the need for invasive ventilation [2-4] and reduce hospital mortality [5]. Noninvasive bi-level positive airway pressure (BiPAP) ventilation delivers positive airway pressure at two different levels during inspiration and expiration, and can decrease inspiratory work of breathing more than CPAP can alone [6]. Studies evaluating BiPAP in acute cardiogenic pulmonary oedema have shown that it improves gas exchange [7] and reduces the need for invasive ventilation in patients with hypercapnic respiratory failure compared with conventional medical therapy [8]. However, none of these studies demonstrated a reduction in hospital mortality [5]. Furthermore, the results of one of the earlier studies suggested that BiPAP compared with CPAP might increase the risk for new onset acute myocardial infarction in patients with acute cardiogenic pulmonary oedema [9]. Whether BiPAP is advantageous compared with CPAP in acute cardiogenic pulmonary oedema remains uncertain.

In the present meta-analysis we assessed the potential beneficial and harmful effects of BiPAP compared with CPAP in patients with acute cardiogenic pulmonary oedema. We also assessed whether the BiPAP may be more advantageous when the pressure used during BiPAP is titrated according to clinical need and in the subgroup of patients with significant hypercapnia (mean arterial carbon dioxide tension [PaCO_2_] > 45 mmHg).

## Materials and methods

A literature search was conducted using the Cochrane Controlled Trials Register (2005 issue 3), and EMBASE and MEDLINE databases (1966 to 1 December 2005). Only randomized controlled clinical trials comparing BiPAP with CPAP in patients with acute cardiogenic pulmonary oedema were included. Studies comparing BiPAP with CPAP in a heterogeneous group of patients with different causes of acute respiratory failure were excluded unless outcomes data for the subgroup of patients with acute cardiogenic pulmonary oedema were available. Studies using both CPAP and BiPAP in the same group of patients in a crossover design were excluded because the clinical outcomes as a result of a particular treatment modality could not be ascertained. During the electronic database search, the following exploded MeSH terms were used: 'bilevel', 'pressure support', 'non-invasive', 'CPAP', or 'positive pressure', with 'ventilation' or 'support' and with 'pulmonary oedema', 'cardiac failure', 'heart failure', or 'respiratory failure'. The reference lists of related reviews and original articles identified were searched for relevant trials. Finally, the websites of the International Network of Agencies of Health Technology Assessment and International Society of Technology Assessment in Health Care were searched to ensure that all suitable studies were included. The authors of one study were contacted to obtain additional information but they did not respond to the request. No studies published in languages other than English were found in the literature search.

Two independent reviewers examined the titles and the abstracts of all identified trials to confirm that they fulfilled the inclusion criteria. The same reviewers examined and recorded the trial characteristics and outcomes independently, using a pre-designed data abstraction form. This abstraction form was used to record information regarding the quality of the trial, such as allocation concealment, randomization method, blinding of treatment, and inclusion and exclusion criteria. The quality of the study was scored according to the Jadad scale (range from 0 to 5, with higher scores indicating better quality) [10], but the individual component that constitutes the quality of the study was also described. The grading of allocation concealment was based on the Cochrane approach (i.e. adequate or uncertain or clearly inadequate). Blinding of the attending physician who decided when to initiate invasive ventilation or to cease noninvasive ventilation with the assigned mode of noninvasive ventilation (for instance CPAP or BiPAP) was required for a study to qualify as double blind. Any disagreements between the two independent reviewers were resolved by consensus. Data were checked and entered into the Review Manager (version 4.2.6 for Windows, 2003; The Cochrane Collaboration, Oxford, UK) database for further analysis.

The hospital mortality and the proportion of patients requiring invasive ventilation (or intubation) were chosen as main outcomes of this meta-analysis because they are the most relevant clinical outcomes of noninvasive ventilation in patients with acute cardiogenic pulmonary oedema. The criteria for requiring invasive ventilation varied between studies, but the common criteria included poor state of consciousness (Glasgow Coma Scale score ≤ 13), respiratory arrest or significant respiratory distress (respiratory rate >40 breaths/minute), persistent hypoxaemia despite supplementary oxygen (arterial oxygen tension <60 mmHg) and progressive increases in PaCO_2 _despite CPAP or BiPAP treatment (>5 mmHg). The other outcomes assessed in the meta-analysis included the proportion of patients who developed new onset acute myocardial infarction after initiation of BiPAP or CPAP, duration of noninvasive ventilation needed till the pulmonary oedema resolved, and length of hospital stay. The criteria and process of weaning from CPAP or BiPAP varied between studies, but the common criteria included absence of respiratory distress with respiratory rate below 25 breaths/minute and pulse oximetry saturation of 95% or greater. The weaning process usually involved stepwise reduction in inspiratory and expiratory pressures (2 cmH_2_O) and inspired oxygen concentration (10%).

### Statistical analyses

The differences in categorical outcomes between the treatment and placebo group were reported as relative risk (RR) with 95% confidence interval (CI), using a random effect model. The effects of CPAP or BiPAP on the hospital mortality and the need for invasive ventilation were further stratified into studies using either a fixed level or variable levels of airway pressure during BiPAP treatment, and this interaction was tested by relative risk ratio [11]. The differences in the duration of noninvasive ventilation required until the pulmonary oedema resolved and the length of hospital stay were reported as weighted mean differences (WMDs), using a random effect model. The presence of heterogeneity between trials was assessed using the χ^2 ^statistics and the extent of inconsistency was assessed using *I*^2 ^statistics [12]. Because hypercapnia is a predictor of requiring intubation in patients with cardiogenic pulmonary oedema [13], sensitivity analysis was conducted to include studies that involved patients with hypercapnia (mean PaCO_2 _> 45 mmHg) before initiation of either CPAP or BiPAP. Publication bias was assessed by funnel plot using hospital mortality as an end-point.

## Results

**Figure 1:**
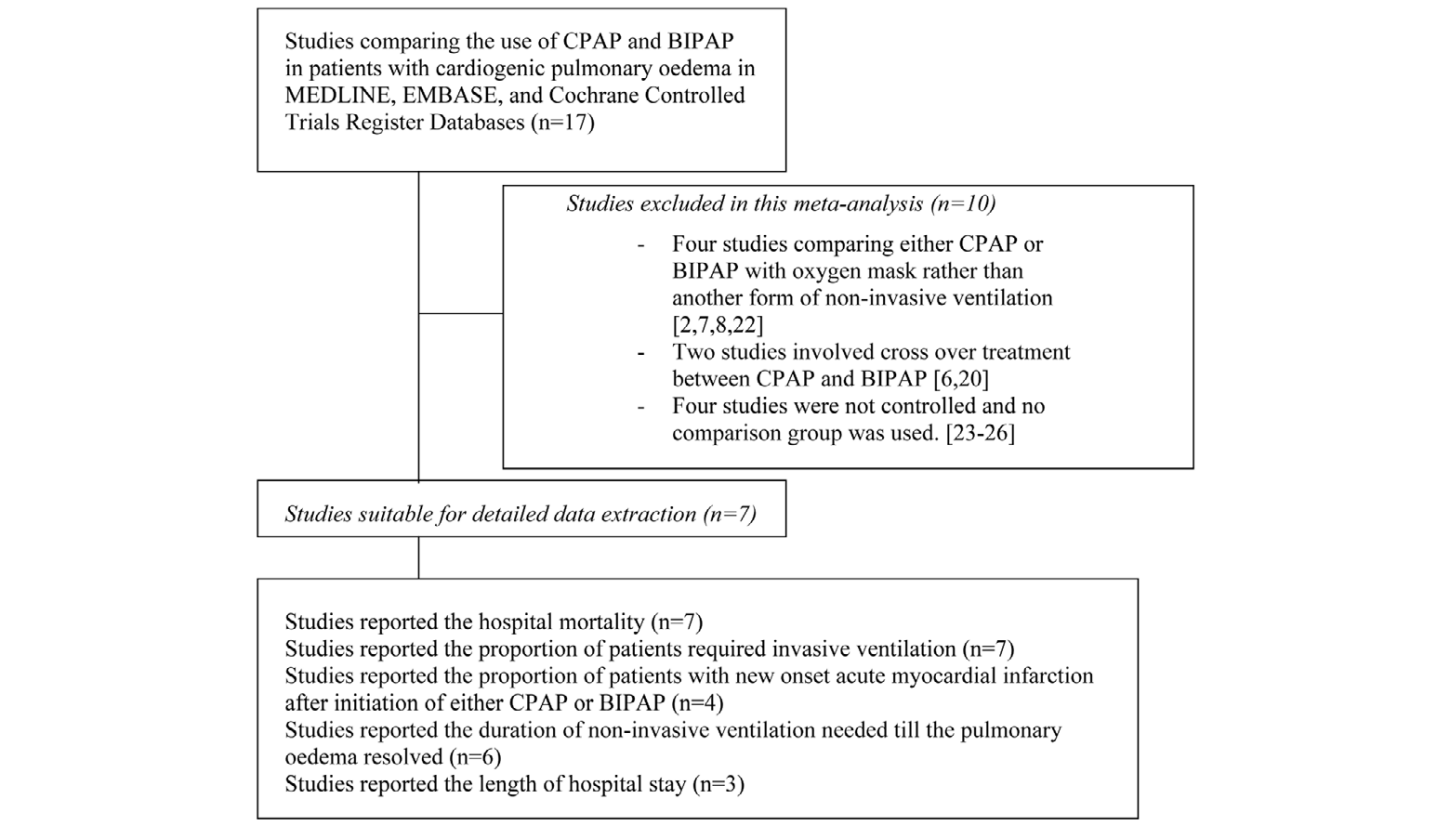
Flow chart: study inclusion and exclusion in the meta-analysis. BiPAP, bi-level positive airway pressure; CPAP, continuous positive airway pressure.

We identified 17 potentially eligible studies, of which seven studies [9,14-19], including a total of 290 patients, fulfilled the inclusion criteria and were subjected to meta-analysis (Figure 1). Two studies used fixed level of CPAP (10 cmH_2_O) and BiPAP (15 and 5 cmH_2_O), two studies used a fixed level of CPAP (10 cmH_2_O) but titrated the level of peak inspiratory pressure in the BiPAP group (from 15 cmH_2_O to achieve a tidal volume of 400 ml), and three studies titrated the level of both CPAP (5–20 cmH_2_O) and BiPAP (peak inspiratory pressure range: 10–25 cmH_2_O). Four studies [9,17-19] recruited patients who presented with hypercapnia (mean PaCO_2 _>45 mmHg). The mean age of the patients ranged from 61 to 77 years and the mean Acute Physiology and Chronic Health Evaluation II scores ranged from 17 to 20 in the pooled studies. The Jadad scale scores of the studies ranged from 2 to 5 (mean 3). Allocation concealment was clearly adequate in four studies but only one study used double blinding. The study details are described in Table 1.

**Figure 2:**
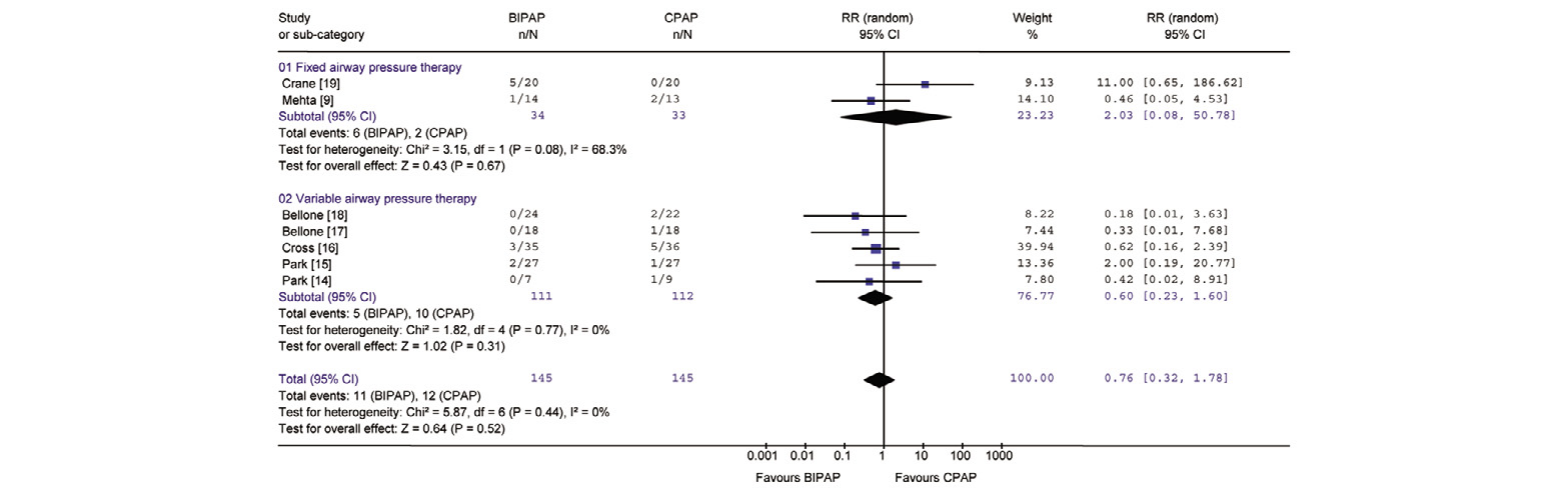
Forest plot: effect of BiPAP and CPAP on hospital mortality. BiPAP, bi-level positive airway pressure; CI, confidence interval; CPAP, continuous positive airway pressure; RR, relative risk.

**Figure 3:**
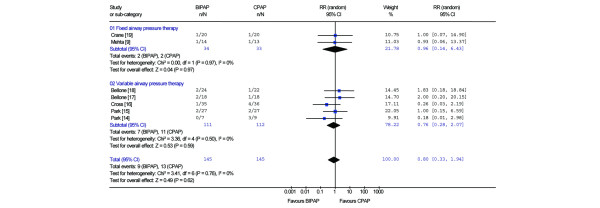
Forest plot: effect of BiPAP and CPAP on risk for requiring invasive ventilation. BiPAP, bi-level positive airway pressure; CI, confidence interval; CPAP, continuous positive airway pressure; RR, relative risk.

There was good overall consistency in most of the results, without significant heterogeneity. Hospital mortality (RR 0.76, 95% CI 0.32–1.78; *P *= 0.52; *I*^2 ^= 0%) and the risk for requiring invasive ventilation (RR 0.80, 95% CI 0.33–1.94; *P *= 0.62; *I*^2 ^= 0%) were not significantly different between patients treated with CPAP and those treated with BiPAP (Figures 2 and 3). Stratifying studies into use of BiPAP at variable or fixed level of airway pressure did not change the results. The relative risk ratios for hospital mortality and requiring invasive ventilation between studies using BiPAP at a fixed level and studies titrating the pressure were 3.38 (95% CI 0.30–37.60; *P *= 0.32) and 1.26 (95% CI 0.15–10.94; *P *= 0.83), respectively. Sensitivity analysis including studies that involved patients with significant hypercapnia did not change the results.

**Figure 4:**
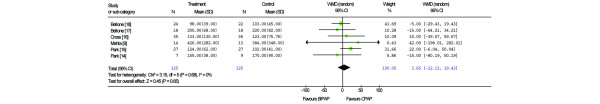
Forest plot: effect of BiPAP and CPAP on duration of noninvasive ventilation needed to resolve pulmonary oedema. BiPAP, bi-level positive airway pressure; CI, confidence interval; CPAP, continuous positive airway pressure; WMD, weighted mean difference.

**Figure 5:**
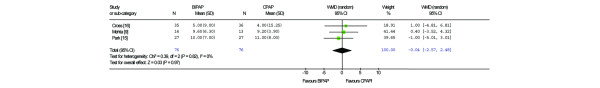
Forest plot: effect of BiPAP and CPAP on length of hospital stay. BiPAP, bi-level positive airway pressure; CI, confidence interval; CPAP, continuous positive airway pressure; WMD, weighted mean difference.

**Figure 6:**
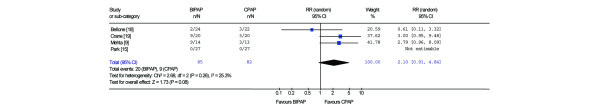
Forest Plot: effect if BiPAP and CPAP on risk of new onset myocardial infarction. BiPAP, bi-level positive airway pressure; CI, confidence interval; CPAP, continuous positive airway pressure; RR, relative risk.

The duration of noninvasive ventilation needed until pulmonary oedema resolved (WMD in hours = 3.65, 95% CI -12.12 to +19.43; *P *= 0.65; *I*^2 ^= 0%) and the length of hospital stay (WMD in days = -0.04, 95% CI -2.57 to +2.48; *P *= 0.97; *I*^2 ^= 0%) were not significantly different between the two groups (Figures 4 and 5). Data on patients with new onset acute myocardial infarction after initiation of CPAP or BiPAP were limited and with some inconsistencies. Based on these limited data, there was an insignificant trend toward an increase in new onset myocardial infarction in patients treated with the BiPAP (RR 2.10, 95% CI 0.91–4.84; *P *= 0.08; *I*^2 ^= 25.3%; Figure 6). None of the studies included a cost-effectiveness analysis.

## Discussion

### Significance of the findings

The present meta-analysis indicates that BiPAP has no significant clinical advantage over CPAP in terms of reducing hospital mortality, requirement for invasive ventilation, length of noninvasive ventilation needed until resolution of pulmonary oedema, and hospital length of stay in patients with acute cardiogenic pulmonary oedema. Based on the limited data available, BiPAP was associated with a trend toward increased risk for new onset acute myocardial infarction compared with CPAP.

BiPAP can reduce work of breathing more than CPAP can in patients with acute cardiogenic pulmonary oedema [6]. Titrating the level of airway pressure used during BiPAP to a targeted tidal volume or to the patient's clinical needs may render BiPAP more beneficial in patients with hypercapnic acute cardiogenic pulmonary oedema because it reduces the work of breathing more effectively than does CPAP while avoiding unnecessary airway pressure that can cause an excessive reduction in cardiac output [8]. However, we were unable to demonstrate any significant clinical benefit of BiPAP over CPAP, including in the subgroup of patients who received titrated BiPAP support and in patients with significant hypercapnia. The reasons for the lack of benefit from BiPAP as compared with CPAP in patients with acute cardiogenic pulmonary oedema remain uncertain.

If we assume that the proportion of patients who may require invasive ventilation or intubation in acute cardiogenic pulmonary oedema is 10%, then the sample size of this meta-analysis (*n *= 290) could only achieve a positive significant result if the relative risk reduction for requiring intubation is more than 80% when BiPAP is compared with CPAP. On the other hand, if the relative risk reduction for requiring intubation after use of BiPAP is more modest (for example, 40%), then a sample size of more than 600 patients would be needed to demonstrate such a difference. Therefore, it is still possible that BiPAP is superior to CPAP in acute cardiogenic pulmonary oedema but that this meta-analysis was underpowered to detect such a modest effect. A large randomized controlled study is needed to confirm whether BiPAP is equivalent or superior to CPAP in patients with acute cardiogenic pulmonary oedema.

The proportion of patients with new onset myocardial infarction was reported in four studies but there were some inconsistencies in the results [9,15,18,19]. Two studies reported an insignificant increase in risk for new onset acute myocardial infarction and two studies did not show such an effect [15,18]. Pooling the limited data together from these four studies resulted in an insignificant trend toward an increase in risk for new onset myocardial infarction. However, patients recruited in these studies were, by nature, at high risk for developing acute myocardial infarction, either before or during the early phase of hospitalization. The small total number of patients included in these four studies (*n *= 167) could have generated a trend toward a false-positive result caused either by a small imbalance in baseline characteristics of the patients or by just a random effect. Nevertheless, use of excessive airway pressure in CPAP or BiPAP has been demonstrated to reduce cardiac output, especially when the left atrial filling pressure is less than 12 mmHg [6,20]. When BiPAP was compared with conventional medical therapy (without CPAP) in two moderate size randomized controlled studies, there were no significant differences in the incidence of new onset acute myocardial infarction [8,15]. On the other hand, when BiPAP was compared with high-dose intravenous isosorbide-dinitrate in acute cardiogenic pulmonary oedema, there was a significant increase in myocardial infarction in patients treated with BiPAP [21]. Therefore, a small increase in risk for reducing cardiac output and/or inducing myocardial ischaemia with BiPAP cannot completely be excluded.

Cost-effectiveness analyses were not reported in any of the pooled studies. A CPAP machine is, in general, cheaper than a BiPAP machine [15]. Based on the lack of significant clinical benefit identified in this meta-analysis, use of BiPAP instead of CPAP is unlikely to be cost-effective in patients with acute cardiogenic pulmonary oedema. Formal cost-effectiveness analysis should be considered if a large randomized controlled study comparing BiPAP and CPAP in patients with acute cardiogenic pulmonary oedema is planned.

### Limitations of the study

Meta-analyses are prone to bias. The quality of trials can affect the direction and magnitude of treatment effect in such analyses. Although most of the included studies had low patient attrition and a Jadad scale score of 3 or higher, some degree of double blinding was attempted only in one study by covering the control panel of the noninvasive ventilator [9]. The physicians who decided when to initiate invasive ventilation or to cease noninvasive ventilation were not blinded to the assigned mode of noninvasive ventilation in the other six studies, and therefore bias might have affected the results. Future noninvasive ventilation studies should consider blinding the attending physician to the mode of noninvasive ventilation used.

**Figure 7:**
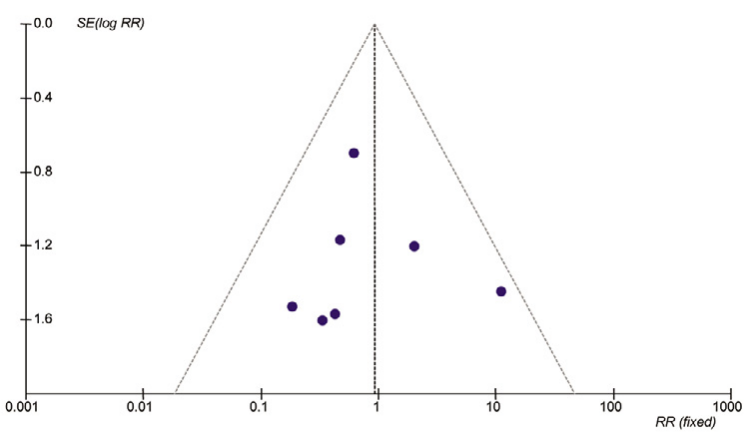
Funnel plot showing the possibility of a small publication bias. RR, relative risk; SE, standard error.

Publication bias can affect the direction and magnitude of the results of a meta-analysis. The funnel plot showed that there might be a small degree of publication bias, with the possibility of missing two small studies (Fig. 7). Nevertheless, this small potential bias was unlikely to change the significance and direction of the results of the meta-analysis.

## Conclusion

Based on the limited data available, BiPAP does not appear to offer any significant clinical benefits over CPAP in patients with acute cardiogenic pulmonary oedema. Until a large randomized controlled trial can demonstrate that BiPAP is associated with significant clinical benefit or is more cost-effective than CPAP in patients with acute cardiogenic pulmonary oedema, the choice of modality will depend mainly on the equipment available.

## Key messages

• 	BiPAP does not offer any significant clinical benefit over CPAP in patients with acute cardiogenic pulmonary oedema.

• 	Until a large randomized control trial can show that BiPAP is associated with significant clinical benefits or is more cost-effective than CPAP in patients with acute cardiogenic pulmonary oedema, the choice of modality will depend mainly on the equipment available.

## Abbreviations

BiPAP = bi-level positive airway pressure; CI = confidence interval; CPAP = continuous positive airway pressure; RR = relative risk; WMD = weighted mean difference.

## Competing interests

The authors declare that they have no competing interests.

## Authors' contributions

KMH had the original idea for the study, conducted data extraction and statistical analyses, and drafted the manuscript. KW conducted data extraction and helped to draft the manuscript. Both authors read and approved the final manuscript.

## Figures and Tables

**Table 1:** Characteristics of the included studies

Reference	Definition of failed noninvasive ventilation	Participants	Interventions	Outcomes	Factors pertaining to the quality of studies
[15]	GCS score ≤13, persistent severe respiratory distress, Po_2 _<60 mmHg despite oxygen supplementation, an increase in PaCO_2 _>5 mmHg	56 patients (recruited between January 1999 and August 2000), mean APACHE II score 19.5, mean age 64 years, mean PaCO_2 _at presentation 40 mmHg	Variable CPAP (10–16 cmH_2_O), variable BiPAP (starting from 15 and 10 cmH_2_O; maximum not described)	Proportion of patients with hospital mortality and requiring intubation, the duration of noninvasive ventilation needed and hospital length of stay	Allocation concealment adequate; study not blinded; 3.6% of patients did not have pulmonary oedema were excluded from analysis; analysis was by intention to treat; Jadad scale score 3
[9]	Severe respiratory distress, inability to tolerate or synchronize with the mask, further deterioration in vital signs or significant haemodynamic compromise, Po_2 _<60 mmHg despite oxygen supplementation, an increase in PaCO_2 _>5 mmHg	36 patients, mean APACHE II score 18, mean age 77 years, mean PaCO_2 _at presentation 50 mmHg	Fixed CPAP (10 cmH_2_O), fixed BiPAP (15 and 5 cmH_2_O)	Proportion of patients with hospital mortality and requiring intubation, new onset acute myocardial infarction, duration of noninvasive ventilation needed and hospital length of stay	Allocation concealment not clear; double blinding; 22% of enrolled patients did not have pulmonary oedema and were excluded from analysis, and 2.8% were excluded because of delay in starting CPAP; analysis was not by intention to treat; Jadad scale score 5
[14]	Intubation determined by the attending physician but criteria were not described	16 patients (recruited between May and October 1997), mean APACHE II score not available, mean age 69 years, mean PaCO_2 _at presentation 40 mmHg	Variable CPAP (5–12.5 cmH_2_O), variable BiPAP (starting from 8 and 3 cmH_2_O; maximum not described)	Proportion of patients with hospital mortality and requiring intubation, duration of noninvasive ventilation needed	Allocation concealment not clear; not blinded; no loss to follow up; analysis by intention to treat; Jadad scale score 2
[16]	Respiratory arrest, respiratory pauses with loss of consciousness, agitation making nursing care impossible and requiring sedation, haemodynamic instability with systolic blood pressure <70 mmHg, and the clinician could elect to intubate if they felt that the patient's condition was not improving satisfactorily	71 patients, mean APACHE II score not available, mean age 74 years, mean PaCO_2 _at presentation not available	Variable CPAP (5–20 cmH_2_O), variable BiPAP (starting from 10 and 5 cmH_2_O; maximum 20 and 5)	Proportion of patients with hospital mortality and requiring intubation, the duration of noninvasive ventilation needed and hospital length of stay	Allocation concealment not clear; not blinded; no loss to follow up; analysis by intention to treat; Jadad scale score 2
[18]	Respiratory arrest, respiratory pauses with loss of consciousness, agitation making nursing care impossible and requiring sedation, haemodynamic instability with systolic blood pressure <70 mmHg	46 patients (recruited between March 2002 and March 2003), mean APACHE II score 18, mean age 77 years, mean PaCO_2 _at presentation 54 mmHg	Fixed CPAP (10 cmH_2_O), variable BiPAP (starting with 15 and 5 cmH_2_O; titration of inspiratory pressure to achieve tidal volume >400 ml)	Proportion of patients with hospital mortality and requiring intubation, new onset acute myocardial infarction, duration of noninvasive ventilation needed	Allocation concealment adequate; not blinded; no loss to follow up; analysis by intention to treat; Jadad scale score 3
[17]	Respiratory arrest, respiratory pauses with loss of consciousness, agitation making nursing care impossible and requiring sedation, haemodynamic instability with systolic blood pressure <70 mmHg	36 patients (recruited between January 2001 and January 2002), mean APACHE II score 18, mean age 77 years, mean PaCO_2 _at presentation 63 mmHg	Fixed CPAP (10 cmH_2_O), variable BiPAP (starting with 15 and 5 cmH_2_O; titration of inspiratory pressure to achieve tidal volume >400 ml)	Proportion of patients with hospital mortality and requiring intubation, duration of noninvasive ventilation needed	Allocation concealment adequate; not blinded; no loss to follow up; analysis by intention to treat; Jadad scale score 3
[19]	Worsening clinical signs (respiratory rate >40 or <10 breaths/minute, reduced conscious level) associated with a falling arterial pH (less than at arrival and <7.2)	40 patients, mean APACHE II score not available, mean age 75 years, mean PaCO_2 _at presentation 62 mmHg	Fixed CPAP (10 cmH_2_O), fixed BiPAP (15 and 5 cmH_2_O)	Proportion of patients with hospital mortality and requiring intubation, new onset myocardial infarction	Allocation concealment adequate; not blinded; no loss to follow up; analysis by intention to treat; Jadad scale score 3

APACHE, Acute Physiology and Chronic Health Evaluation; BiPAP = bi-level positive airway pressure; CPAP = continuous positive airway pressure; GCS, Glasgow Coma Scale; PCO_2_, partial carbon dioxide tension; Po_2_, partial oxygen tension.
